# A Peculiar Restoration

**Published:** 1899-09

**Authors:** Ellison Hillyer

**Affiliations:** Brooklyn.


ARTICLE VIII.

### A PECULIAR RESTORATION.

ELLISON HILLYER, D. D. S., BROOKLYN.

The crowning of an upper lateral incisor is an easy matter where conditions exist which warrant the cutting off of the tooth and the usual procedure of root trimming, concluding with the usual crowning.

When a case presents which requires restoration, such as the following, some study of the case is necessary to meet the required success. It is with this thought in mind that I take up the description of this case, trusting it may be of benefit to some one.

The case presented a perfectfy formed upper arch with the exception of the lateral incisors; the right lateral was missing with no space between the central incisor and the canine; the left lateral was a mere peg-shaped tooth of nearly the proper length, but in other respects undeveloped (as far as shape is concerned). To restore this to proper shape and appearance was the object.

The patient, a young lady and a singer, felt the effect of the appearance of her mouth when showing her teeth, and the result was an habitual dropping of the lip upon the left side in the endeavor to cover the defect. A further object, therefore, was to correct this habit.

The tooth, peg shaped though it was, was fully alive, with perfectly formed enamel and no cavities. The plan of destroying the pulp and utilizing the canal did not appear advisable, nor did I desire to cut off the tooth. I finally decided to proceed without disturbing the existing conditions.

As the recital of failure is often as valuable a guide as of success in warning how not to proceed, and as ultimate success crowned my efforts in this case, I will tell how the first attempt did not succeed and why.

After making a die and counter-die and swaging a perfect fitting cap for the tooth of thin platinum, I selected a lateral tooth suitable as to size and color; then without regard to the pins I ground the tooth to fit over the labial aspect of the cap in position upon the tooth. This completed, I baked the two (cap and porcelain tooth) together with continuous gum body. When cemented upon the tooth, the condition appeared quite satisfactory for a short time, at the expiration of which the patient returned with the facing bitten off. The conclusion was that the porcelain was not sufficiently strong in itself to withstand the strain. So much for the failure.

Proceeding with a capping of platinum similar to the first, I selected a cuspid tooth about twice the required length, or at least long enough to cover the peg-shaped tooth with the porcelain above the pins, I then cut off the remaining portion of the porcelain tooth below the line of the pins and ground to fit the capping, as before, but preserving the pins. I used in this instance a cuspid tooth for two reasons; first, to match the opposite cuspid (the right lateral being missing), and, second, to allow a little extra support of porcelain to the pins by the pointing of the tooth. The appearance of this tooth was better than the first.

With the porcelain tooth in position against the platinum capping, I bent up the pins on each side of the capping and soldered with pure gold, subsequently covering the back with continuous gum body as before. This, when set upon the peg-shaped tooth, restored the part satisfactorily, and has given active service for about a year, and seems good for prolonged use.—*Items of Interest.*

